# Evaluating implementation of WHO Trauma Care Checklist vs. modified WHO checklist in improving trauma patient clinical outcomes and satisfaction

**DOI:** 10.5249/jivr.v13i1.1579

**Published:** 2021-01

**Authors:** Shahram Bidhendi, Alireza Ahmadi, Mona Fouladinejad, Shahrzad Bazargan-Hejazi

**Affiliations:** ^*a*^ Department of Anesthesiology, Critical & Pain Management, Kermanshah University of Medical Sciences, Kermanshah, Iran.; ^*b*^ College of Medicine, Charles R. Drew University of Medicine and Science, CA, U.S.A.

**Keywords:** WHO Trauma Care Checklist, Pain Management, Patient Satisfaction, Trauma Care Management, WHO Modified Trauma Care Checklist

## Abstract

**Background:**

Use of checklist in evaluation of trauma patients has been a critical component of improving the care process and reducing medical errors and increasing patient's quality of life. We aim to assess the impact of the modified World Health Organization Trauma Care Checklist (WHO TCC) on the management of pain, complications, mortality and patient satisfaction in trauma patients.

**Methods:**

This was a randomized control trial (RTC). Trauma patients referred to the trauma center and met the eligibility criteria were randomly assigned into three study groups. Group 1 were patients who received trauma care without using the WHO checklist, and only by the standard of care. Group 2 were patients who received trauma care according to the WHO's checklist, and group 3 were patients received trauma care according to the WHO's modified checklist. We used independent t-test and chi-square tests to assess the association between the study variables with checklist groups. The significance level of tests was set for p-value less than 0.05.

**Results:**

We observed patients’ level of pain, Injury Severity Score (ISS), Glasgow Coma Criterion (GCS) and patient satisfaction significantly improved across the checklist groups, but more so in the modified checklist group (P less than 0.001). Similarly, findings reveal significant relationships between all clinical characteristics of the patients and checklist groups, except for a CT Scan of the spinal cord. We were unable to establish any significant associations between the checklist groups and the majority of the selected trauma care process measures, except for missed injury (p = 0.001).

**Conclusions:**

Both the WHO TCC and the WHO modified checklist, in the initial assessment and during the treatment and care processes, enhance patients’ clinical outcomes. However, patients in the modified checklist compared to WHO TCC reported a higher level of satisfaction. Implications and future directions are discussed.

## Introduction

Trauma is any wound or penetrating or non-penetrating injury caused intentionally or unintentionally by external factors in the human body.^1^ Trauma injury is one of the leading causes of death and disability, responsible for more deaths than deaths due to HIV, malaria, and tuberculosis combined.^2^ Globally, trauma injury burdens countries at all levels of development mentally, socially, and economically.^3^ In Iran, trauma is the second leading cause of premature death in the young population, regardless of gender.^3-5^

In patients with severe trauma, the primary goal is patient survival, and the secondary goals are avoiding organ failure, other complications, speeding up recovery, and ultimately achieving the desired quality of life.^6^ Therefore, early systematic evaluation of trauma patients is a critical component of improving the care process, reducing medical errors, and increasing patients’ quality of life.^7^ The efficacy of checklist implementation to improve patient safety, optimize care, and reduce medical errors has been reported airway management, fluid resuscitation, and diagnosis of life-threatening injuries.^8-13^

**WHO checklist**

The WHO Trauma Care checklist (TCC) is a simple tool that is designed to ensure the safety of trauma patients in life-threatening conditions.^14^ TCC identifies minimum sets of steps taken in care of all trauma patients admitted in emergency units, regardless of resource availability.^15^ It is designed to standardize and reinforce aspects of early assessment of patients with trauma, thereby reducing the likelihood of diagnostic, therapeutic, and care errors during initial resuscitation.^15^ TCC validity has been tested by global collaboration across different emergency units.^14^

The WHO TCC consists of two main sections. The first section of the checklist includes immediate and urgent activities that should be followed right after the primary and secondary examinations, which involve eleven steps. Steps include 1) assessing if airway intervention is needed, 2) evaluation for tension pneumo-haemothorax, 3) check if the oximetry pulse is placed and functioning, 4) check of large-bore IV and liquid has started, 5) conduct complete assessments for and control of external bleeding, 6) evaluation for any pelvic fracture, 7) evaluation for any internal bleeding, 8) assess if spinal immobilization is needed, 9) check the neurovascular status of four limbs, 10)assess if the patient is hypothermic, 11) evaluate for other patient needs (if no contraindication). 

The second part of the WHO TCC includes five steps that should be followed before the medical team could leave the patient. Step 1: Has the patient been given the prescribed medications? Step 2: Have all lab tests and imaging been reviewed? Step 3: Has it been identified which serial examinations are needed? Step 4: Has patient's treatment plan discussed with the patient or the assigned representative, and step 5: Has the patient’s charts that are related to the trauma been completed?

Results of recent studies show that WHO's checklist for trauma care reduces mortality,^16^ delivers favorable treatment results,^17^ and improves patient self-report of the treatment outcome.^18^ Although the WHO checklist has been useful in coordinating and harmonizing trauma care and services, the checklist is short of providing the critical steps for the management of pain in trauma care. Therefore, due to the vital role of pain management in patients, in the current study, we added ‘pain management’ as an additional step to the first part of the checklist. Hereafter we call the modifiedchecklist “WHO modified checklist”. The pain management items include assessing patient’s pain intensity and prescribing medications according to the level of pain, as indicated below.

**Suggested Pain Management Evaluation for Trauma Patients**

**Table:** 

Pain Intensity	Resulting score	Prescribe drug
Mild	1-3	Pentazocine / ketorolac
Moderate	4-6	Tramadol and Pethidine
Intense	7-10	Morphine / Fentanyl

We aim to assess the impact of the modified World Health Organization Trauma Care Checklist (WHO TCC) on the management of pain, complications, mortality and patient satisfaction in trauma patients.

## Methods 

This was a randomized control trial (RTC). The patient population included all trauma patients referred to the trauma center of Ayatollah Taleghani Hospital in Kermanshah, the research site. To be eligible in the study, the research sample had to have the following characteristics:

*Inclusion criteria*

1. Age between 18 and 60 years old 

2. Glasgow Coma Scale (GCC) equal to or more than 10

3. Sustain life-threatening damage to an internal organ(s) determined by the clinical judgment of the treating physician

4. No pregnancy

5. No history of chronic mental illness, lung or kidney disease

6. Not undergoing chemotherapy.

7. No illicit drug dependency

8. Consenting to participate in the study

Patients who did not meet the inclusion criteria were excluded from the study participation. Also, during the study process, the study principal investigator excluded patients who refrained from continuing the study and those with the incomplete checklist.

**Sampling method and sample size**

We used a computer-generated random sample of patients from the list of eligible patients. We determined the sample size based on considering that the relative percentage of improvement in the 19 indicators of the WHO checklist is 25% in the cases were the checklist was used compared to cases where the checklist was not used.^15^ We calculated the sample size using a minimum reliability coefficient of 95% and a power of 80%, which led to a sample of 60 patients for each of the three study groups; WHO checklist, modified WHO checklist, and no checklist. 

**Assignment to the treatment groups**

After obtaining study approval from the ethics committee of Kermanshah University of Medical Science (KUMS) in a period of three months in 2018, patients who were referred to the trauma center in Ayatollah Taleghani Hospital in Kermanshah, and met the eligibility were randomly assigned into three study groups.

Group 1: Patients who received trauma care without using the WHO checklist, and only by the standard of care. Group 2: Patients who received trauma care according to the WHO's checklist. Group 3: Patients received trauma care according to the WHO's modified checklist.

During the study, the pain intensity of patients with numerical scale was calculated, and therapeutic interventions were performed. Patients were treated for one month and then assessed for pain severity, the severity of the injury, treatment received, mortality rate, and complications post-trauma complications. Patients were discharged from the hospital and were followed on as needed basis either by phone or face-to-face. We obtained the approval of the ethics committee of Kermanshah University of Medical and Sciences to conduct the study. 

**Data collection tools**

*We completed demographic *information through a direct interview with the patient or patient’s companion or using the information in their medical chart. Demographic information includes gender, age, education, marital status, and place of residence.

*Assessment of the severity of the injury: *Three researchers in the current study received training regarding the calculation of the Injury Severity Score (ISS) to ensure standardized scoring across their checklist evaluation. The ISS scale measures the severity of the injury on a scale of zero to 75. To examine the extent of trauma, we used a typical trauma scale ranging from a score of 1, meaning a mild injury, and a score of 6, meaning a lethal injury (2=moderate injury, 3=serious injury, 4=severe injury, 5=critical injury, and 6=fatal injury) for any of the face, chest, abdomen, limbs, and external surfaces. To estimate the ISS, the squared of the Abbreviated Injury Scale (AIS) on the three most damaged areas were calculated and summed. 

*Pain intensity assessment scale: *This scale has been used in various studies, and its reliability has been reported (a = 0.94).^19^ Patient self-report of pain intensity was assessed by asking a patient to indicate the amount of pain experienced on a scale of zero to ten on a ten-centimeter calibrated line, where zero indicates no pain and ten means the maximum intolerable pain.

*Mortality: *We estimated mortality by dividing the number of injured patients who participated in this study and divided by the total number of injured patients multiplied by 100. 

*Medical chart data: *Using patient's medical chart we recorded and monitored patient's critical clinical data and medical histories, such as vital signs, diagnoses, medications, physical and radiological examinations, data in the patient's medical chart, the status of clinical examinations, radiological images, laboratory and test results. We also recorded complications from trauma, including cardiac arrest, pneumonia, pulmonary embolism, renal failure, sepsis, septic shock, wound infection, and more. 

*Complications: *This information was extracted from the patient chart and included cardiac arrest, pneumonia, pulmonary embolism, renal failure, sepsis, septic shock, wound infection, etc.

**Data analysis**

We used STATA software for data analysis. In addition to reporting descriptive statistics, we used independent t-test and chi-square tests to assess the association between the study variables with checklist groups. The significance level of tests was set for p-value<0.05.

## Results

Sample demographic characteristics are presented in Table 1, which shows there was no significant differences between these variables and the study groups. As illus-trated in Table 2, patients’ level of pain, ISS, GCS, and satisfaction significantly improved across the checklist groups, but more so in the modified checklist (P <0.001). Similarly, findings based on Table 3 reveal that there were significant relationships between all clinical characteristics of the patients and checklist groups, except for CT Scan of spinal cord. We were unable to establish any significant associations between the checklist groups and the majority of the selected trauma care process measures, except for missed injury (p = 0.001) (Table 3).

**Table 1:** Sample characteristics by checklist assignments.

Variable	WHO checklist	Modified WHO checklist	No checklist	P
	N= 60	N= 60	N= 60	
	N(%)	N(%)	N(%)	
**Age **				0.369
Means ± SD	19.8±35.67	19.3±40.31	19.09±39.45	
**Gender**				0.631
Male	39 (65%)	36 (60%)	41 (65.3%)	
Female	21 (35%)	24 (40%)	19 (31.7%)	
**Marital Status**				0.227
Married	33 (60%)	45 (75%)	40 (66.8%)	
Single	22 (40%)	15 (25%)	20 (33.3%)	
**Educational Status**				0.22
Educated	8 (13.3%)	15 (25%)	16 (29.7%)	
Elementary School	18 (30%)	11 (18.3%)	5 (8.3%)	
Junior High	7 (11.7%)	16 (26.7%)	13 (21.7%)	
High School	21 (35%)	13 (21.7%)	20 (33.3%)	
College	4 (6.7%)	2 (3.3%)	6 (10%)	
Unknown	2 (3.3%)	3 (5%)	0 (0%)	
**Employment**				0.001
Self-Employed	17 (29.8%)	16 (26.7%)	15 (25%)	
Government Employee	6 (10.5%)	2 (3.3%)	5 (3.3%)	
Unemployed	17 (29.8%)	3 (5%)	2 (3.3%)	
Student	5 (8.8%)	4 (6.7%)	6 (10%)	
Retired	1 (1.8%)	0 (0%)	0 (0%)	
Manual Labor	1 (1.8%)	5 (8.3%)	12 (20%)	
Unknown	17 (29.8%)	5 (5.3%)	4 (6.7%)	
Homemaker	3 (5.3%)	23 (37.3%)	12 (20%)	
Agriculture	2 (3.5%)	2 (3.3%)	4 (6.7%)	

**Table 2:** Association between the injury characteristics and the checklist groups.

Variable	WHO checklist	Modified WHO checklist	No checklist	P
	N= 60	N= 60	N= 60	
**Level of Pain**				
Means ± SD	3.38 ± 2.2	5.31 ± 2.2	4.31± 2.36	0.001
**Injury Severity Score (ISS)**				
Means ± SD	9.33 ± 5.36	11.41 ± 12.38	9.95 ± 6.56	0.001
**Glasgow Coma Criterion (GCS)**				
Means ± SD	14.03 ± 1.48	14.43 ± 1.29	14.5 ± 2.47	0.003
**Patient Satisfaction **				
Means ± SD	3.95 ±1.3	6.66 ± 0.98	1.1 ± 3.38	0.001

**Table 3:** The associations between selected trauma care process measures and the checklist groups.

Variable	WHO checklist	Modified WHO checklist	No checklist	P
	N= 60	N= 60	N= 60	
**Lung sounds**				0.00
Yes	45 (75%)	15 (25%)	35 (58.3%)	
No	15 (25%)	45 (75%)	25 (41.7%)	
**Pulse Examination**				0.00
Yes	45 (75%)	44 (73.3%)	20 (33.3%)	
No	15 (25%)	16 (26.7%)	40 (66.7%)	
**Physical Examination of Spinal Cord**				0.00
Yes	20 (33.3%)	41 (68.3%)	45 (75%)	
No	40 (66.7%)	19 (31.7%)	15 (25%)	
**Motor Skills Test**				0.00
Yes	42 (70%)	42 (80%)	23 (38.3%)	
No	18 (30%)	18 (30%)	37 (61.7%)	
**Sensory Test**				0.00
Yes	31 (51.7%)	38 (63.3%)	13 (21.8%)	
No	29 (48.3%)	22 (36.7%)	45 (78.3%)	
**Clinical Abdominal Test**				0.00
Yes	45 (75%)	42 (70%)	19 (31.7%)	
No	15 (25%)	18 (30%)	41 (78.3%)	
**Scalp Test**				0.00
Yes	45 (75%)	32 (53.3%)	18 (30%)	
No	15 (25%)	28 (46.7%)	42 (80%)	
**Chest Imaging**				0.00
Yes	42 (70%)	34 (56.7%)	18 (30%)	
No	18 (30%)	26 (43.3%)	42 (70%)	
**Abdominal Ultrasound**				0.003
Yes	14 (23.3%)	21 (51.7%)	14 (23.3%)	
No	46 (76.7%)	29 (47.3%)	36 (76.7%)	
**CT Scan of Spinal Cord**				0.281
Yes	22 (36.7%)	18 (30%)	14 (23.3%)	
No	38 (63.3) %	42 (70%)	46 (76.7%)	
**CT Scan of Abdomen**				0.00
Yes	0 (0%)	15 (25%)	0 (0%)	
No	60 (100%)	45 (75%)	60 (100%)	
**Temperature**				0.01
Yes	37 (61.7)	36 (60%)	19 (31.7%)	
No	23 (38.3)	26 (40%)	41 (78.3%)	
**Tetanus Vaccine Received**				0.935
Yes	31 (51.7%)	33 (55%)	32 (53.3%)	
No	29 (47.3%)	27 (45%)	28 (46.7%)	
**History of Tetanus Vaccine**				0.854
Yes	33 (55%)	30 (50%)	31 (51.7%)	
No	27 (45%)	30 (50%)	29 (45.3%)	
**Counseling Attendance**				0.091
Yes	28 (46.7%)	26 (43.3%)	17 (28.3%)	
No	32 (53.3%)	34 (56.7%)	43 (71.7%)	
**Death**				0.922
Yes	3 (5%)	1 (1.7)	1 (1.7)	
No	47 (95%)	59 (97.3)	59 (98.3)	
**Shock**				0.802
Yes	3 (5%)	1 (1.7%)	3 (5%)	
No	47 (95%)	59 (97.3%)	47 (95%)	
**Tetanus Vaccine Received**				0.935
Yes	31 (51.7%)	33 (55%)	32 (53.3%)	
No	29 (47.3%)	27 (45%)	28 (46.7%)	
**History of Tetanus Vaccine**				0.854
Yes	33 (55%)	30 (50%)	31 (51.7%)	
No	27 (45%)	30 (50%)	29 (45.3%)	
**Heart Failure**				0.366
Yes	0 (0%)	0 (0%)	1 (1.7%)	
No	60 (100%)	60 (100%)	59 (98.3%)	
**Pneumonia **				0.951
Yes	8 (13.3%)	7 (11.7%)	8 (13.3%)	
No	52 (86.7%)	53 (88.3%)	52 (86.7%)	
**Deep Vein Thrombosis**				0.291
Yes	6 (10%)	8 (13.3%)	3 (5%)	
No	54 (90%)	52 (86.7%)	85 (95%)	
**Pulmonary Embolism **				0.807
Yes	3 (5%)	1 (1.7%)	3 (5%)	
No	85 (95%)	59 (98.3%)	85 (95%)	
**Kidney Failure**				0.99
Yes	1 (1.7%)	1 (1.7%)	0 (0%)	
No	59 (98.3%)	59 (98.3%)	60 (100%)	
**Missed Injury**				0.001
Yes	0 (0%)	0 (0%)	5 (11.7%)	
No	60 (100%)	60 (100%)	35 (88.3%)	
**Septic Shock**				0.99
Yes	0 (0%)	0 (0%)	1 (1.7%)	
No	60 (100%)	60 (100%)	59 (98.3%)	
**Sepsis**				0.99
Yes	1 (1.7%)	0 (0%)	1 (1.7%)	
No	59 (98.3%)	60 (100%)	59 (98.3%)	
**Heart Failure**				0.366
Yes	0 (0%)	0 (0%)	1 (1.7%)	
No	60 (100%)	60 (100%)	59 (98.3%)	
**Pneumonia **				0.951
Yes	8 (13.3%)	7 (11.7%)	8 (13.3%)	
No	52 (86.7%)	53 (88.3%)	52 (86.7%)	
**Deep Vein Thrombosis**				0.291
Yes	6 (10%)	8 (13.3%)	3 (5%)	
No	54 (90%)	52 (86.7%)	85 (95%)	

## Discussion

Our study showed that the use of a modified WHO checklist based on pain management in trauma and accident patients is associated with a higher level of patient satisfaction due to the reduction of pain in these patients, compared to the WHO checklist. 

Evaluation of patients showed in the gross sensory test, abdominal ultrasound, and abdomen CT scan use of modified checklist resulted in better evaluation and management of patients compared to patients who were evaluated and treated with the WHO checklist and the group without the checklist. It is possible that the modified checklist has the potential to meet the needs and the condition of the patients, and significantly reduce the incidence of medical error. Similarly, the findings of Ebrahimi and Fakhar study showed that the use of a checklist and standard protocol resulted in better evaluation and treatment of patients. ^20^ However, we did not find any significant difference between the WHO checklist and the modified checklist in evaluation of patients for end pulse test, spinal physical examination, gross motor skill test, abdominal test, temperature assessment, CT scan of spinal cord, history of receiving tetanus vaccine, pneumonia evaluation, and evaluation of vascular thrombosis. But both groups were better off compared with the group that was evaluated without a checklist, which means using a modified checklist or WHO checklist assist the treatment team in evaluating and managing patients. Other studies confirm our findings.^21,22^ The use of checklists and guidelines can effectively guide the treatment team in evaluating patients.^18^ The use of patient evaluation protocols can speed up the action, increase the accuracy of the team in evaluating patients, and ultimately create more appropriate results.^23^

Furthermore, the results showed in the auditory sections, and scalp test patients were assessed by the WHO checklist were better evaluated than other groups, and these results were statistically significant. In the study by Lashour et al.^15^ use of the WHO checklist in patients evaluations resulted in a better outcome. Also, the results showed mortality, the incidence of shock, pulmonary embolism, renal failure, the incidence of septic shock, and sepsis were not significantly different in the patients in any of the three groups. However, in most of these areas, the outcomes observed in the modified checklist group were better. In general, our findings support other studies, which have shown the use of checklist and guideline can improve patient outcomes.^24,25^

**Limitations**

Our study has several limitations, including the probability of using the incomplete recording of information in the patients' files. We tried to compensate for this limitation by training the data abstractors to be consistent, accurate, and objective in extracting information from patient’s chart. Furthermore, this was a single-site study with a small sample. Multisite studies with a larger sample size that include children and older adults (60 and over) are needed to replicate our findings. Additionally, our inclusion criteria limited us to enroll patients with GCS less than 10. Future studies should include patients with low GCS and use behavioral pain scale (BPS)^26^ such as facial expression.^27^

## Conclusion

Both the WHO TCC and the WHO modified checklist, in the initial assessment and during the treatment and care process, enhance patients’ clinical outcomes. However, patients in the modified checklist compared to the WHO TCC reported higher level of satisfaction.
